# The Relationship Between Health Literacy, Autonomic Nervous Activity, and Acceleration Pulse Wave in Japanese Adults: A Cross-Sectional Study

**DOI:** 10.7759/cureus.85802

**Published:** 2025-06-11

**Authors:** Masahiro Noguchi, Ryo Miyachi, Takaaki Nishimura, Akio Goda, Hiromichi Takeda, Eisuke Takeshima, Yuji Kanazawa

**Affiliations:** 1 Department of Physical Therapy, Faculty of Health and Medical Sciences, Hokuriku University, Kanazawa, JPN

**Keywords:** acceleration plethysmography, autonomic nervous system, community-dwelling individuals, health literacy, quality of life

## Abstract

Background: Autonomic nervous activity and acceleration plethysmography (APG) are effective biomarkers for assessing health status. However, the relationship between health literacy and these factors is unclear.

Purpose: This study investigated the relationship between health literacy, autonomic nervous activity, and APG.

Methods: This cross-sectional analysis included 222 Japanese adults. Health literacy was assessed using the Japanese version of the HLS-EU-Q16. Autonomic nervous activity and APG were measured using a pulse analyzer.

Results: Women were significantly older than men (p<0.01) and had higher health literacy (p<0.01). Therefore, health literacy was compared by gender. The high-health-literacy group tended to have a higher age and quality of life, and their APG was significantly higher. Multiple logistic regression analysis showed that high health literacy was associated with age, quality of life, and parasympathetic nerve activity.

Discussion: Improving health literacy in community-dwelling individuals suggests the need for intervention from a younger age. Although the odds ratio was low for both health literacy and autonomic nervous system activity, a correlation was suspected. Further verification is necessary when selecting subjects in the future.

## Introduction

As the Japanese population ages, the gap between the average life expectancy and healthy life expectancy is attracting attention. According to a 2023 Cabinet Office report [1], the difference between average life expectancy and healthy life expectancy for Japanese people was 8.67 years for men and 12.28 years for women in 2003, but by 2019, it had narrowed slightly to 8.73 years for men and 12.07 years for women, with healthy life expectancy increasing slightly compared to average life expectancy. To further extend healthy life expectancy, it is necessary to detect the physical changes that occur before the onset of disease at a younger age. For this purpose, autonomic nervous system activity and Acceleration Plethysmography (APG) tests can be performed to check the condition of the peripheral arteries. The autonomic nervous system regulates body function and plays an essential role in maintaining homeostasis [2]. Autonomic nervous system dysregulation has been shown to underlie numerous cardiovascular diseases, cognitive decline, and age-related health concerns in older adults [3]. The APG test is a useful diagnostic instrument that can noninvasively predict vascular aging and has been identified as a risk factor for atherosclerosis [4]. The utilization of these diagnostic indicators is paramount for the maintenance of optimal health, as it facilitates the timely detection of pathological alterations within the body.

It is imperative to engage in voluntary health actions to maintain optimal health over an extended period without exacerbating health-related indicators. To promote these health actions, it is essential to enhance health literacy. Health literacy is defined as the ability of individuals to locate, comprehend, and apply information and services that are pertinent to their own and others' health-related decisions and actions [5]. Health literacy in adults has been demonstrated to exert a substantial influence on health outcomes. Some studies have reported a correlation between low health literacy and a decline in physical function and mental health, an increase in hospital admissions, an increase in emergency medical care use, and a decline in preventive service use [6,7]. In contrast, existing evidence demonstrates that high health literacy is associated with increased physical activity [8]. In Japan, health literacy has been reported to be significantly associated with the prevalence of multiple chronic diseases, and there have been indications of a link with healthy behaviors in people aged <30 years [9,10]. Consequently, high health literacy may result in health behaviors that maintain healthy autonomic nervous system activity and APG levels, thus preventing future diseases. However, the relationship between health literacy and these factors remains unclear. Therefore, this study aimed to investigate health literacy and its associated factors among adults residing in diverse regions of Japan with a wide age range as a preliminary study to determine whether autonomic nervous system activity and APG are factors that influence health literacy. We hypothesized that poor health literacy is associated with unhealthy autonomic nervous activity or APG status prior to the onset of fatal diseases.

## Materials and methods

Participants

We recruited participants from the general adult population aged ≥20 years who underwent health checkups at workplaces, health education events in the community, and health data measurement events at commercial fitness facilities in Ishikawa Prefecture, Japan, from April to September 2024. The scope of the projects included health checkups for university employees, health education events in Kanazawa City, Ishikawa Prefecture, and health studios in a commercial facility in Komatsu City, Ishikawa Prefecture. Participants were individuals who voluntarily attended these projects and facilities. During the measurement event, the research content, ethical considerations, and publication were explained both verbally and in writing, and if the participant wished to participate, their consent was confirmed by written signature.

Inclusion and exclusion criteria

The inclusion criteria were the ability to reach the research venue independently and the absence of communication barriers. Individuals who could not complete the questionnaire, those with communication barriers, those with cognitive or mental impairments, or those with pacemakers were excluded. The demographic characteristics of the participants were analyzed after the measurements. The collected data were anonymized and stored.

Sample size

The sample size was calculated using G Power [11,12] according to Bujang's steps [13]. However, since the effect size was unknown, it was arbitrarily set to 0.5, and more samples were recruited than the calculation indicated.

Ethical statement

This study was conducted in accordance with the Declaration of Helsinki, and approval was obtained from the Research Ethics Committee of Hokuriku University before the study began (Approval number 2024-1). Written informed consent was obtained from all participants.

Study design

This was a cross-sectional analysis study that aimed to investigate health literacy and its influencing factors in Japanese adults and to clarify the relationship among health literacy, autonomic nervous system activity, and APG.

Data collection

Basic data (date of birth, age, sex, and height) were self-reported data from the participants. Body composition data (body weight, body mass index (BMI), body fat percentage, and skeletal muscle index (SMI)) were measured using a body composition analyzer (InBody 270; InBody Japan Inc., Tokyo, Japan). This device uses bioelectrical impedance analysis (BIA), which involves passing a weak electric current through the body and analyzing the body composition based on resistance.

Questionnaires were administered to survey health literacy and quality of life (QOL). The Health Literacy Survey used the HLS-EU-Q16 Japanese version of the HLS-EU-Q [14]. This survey method is a shortened version of the HLS-EU-Q47 created by a project team commissioned by the European Health Literacy Project Consortium (the HLS-EU Consortium). The HLS-EU-Q16 has also been translated into Japanese, and its validity has also been verified in Japan [15]. The responses to the questionnaire were scored on a 5-point Likert scale: 1. very easy, 2. fairly easy, 3. fairly difficult, 4. very difficult, 5. Do not know/do not apply. Of these five levels, “5. Do not know/not applicable” was treated as a missing value, and the standardized index was calculated from the other response results. The standardized index is calculated on a scale of 0-50, with higher scores indicating better health literacy. In line with the report by the HLS-EU Consortium, scores of 0-24 were judged as “inadequate,” >25-33 as “problematic,” >33-42 as “sufficient,” and >42-50 as “excellent” [14].

The EQ-5D-5L was used to evaluate the QOL. The EQ-5D-5L was developed by the EuroQOL Group as a standardized, non-disease-specific tool for evaluating health-related QOL [16]. This measurement evaluates five aspects-mobility, self-care, usual activities, pain/discomfort, and anxiety/depression. On a scale of 5 levels: no problems, slight problems, moderate problems, severe problems, and extreme problems. The scoring was performed according to the method described by Ikeda et al. [17].

Autonomic nerve balance analysis and acceleration pulse wave measurements were performed using a pulse analyzer (TAS9 VIEW, YKC, Tokyo, Japan). This device measures changes in the volume of peripheral blood vessels as pulse waves (PPG), converts them to acceleration pulse waves (APG), and analyzes peripheral blood circulation (vascular aging) and heart rate variability (HRV) by extracting pulse waves with high precision from the interval between the pulse wave peaks and analyzing their changes. The measurements were obtained in a partitioned space where no outside information could infiltrate while the subjects were seated in a relaxed position with their eyes closed. The measurements were taken automatically by the device; therefore, there was almost no measurement error. APG reflects the aging of blood vessels and shows a high correlation with baPWV, which is an indicator of arteriosclerosis in peripheral blood vessels and is considered a promising test for the early screening of arteriosclerotic diseases [18,19]. The APG can capture cardiac function with four systolic waves and one diastolic wave [18]. In this study, the B ratio, which is the b-wave peak divided by the wave peak, was used. HRV is an indicator of autonomic nervous system activity and reflects psychological stress in healthy adults [20,21]. In HRV measurements, the high-frequency component (HF) reflects parasympathetic nervous system activity, whereas the low-frequency component (LF) reflects sympathetic nervous system activity. The ratio of HF to LF (LF/HF) is an indicator of autonomic nervous system activity [21]. The standard deviation of normal-to-normal intervals (SDNN) is the standard deviation of the variation between heartbeats and represents the overall autonomic nervous activity of the heart, whereas the root mean square of successive differences (RMSSD) represents the parasympathetic regulation of the heart rate. In this study, these indices were used to evaluate the autonomic nervous activity.

Statistical analysis

The data obtained are presented as quartiles and quartile ranges. Owing to the wide age range of the participants, they were divided into six groups: < 40 years old, 40s, 50s, 60s, 70s, and > 80 years old, and the measurements were compared by age group using the Kruskal-Wallis Test. Furthermore, for measurements that showed significant differences, the significance level of the two-group comparison of the Mann-Whitney U test was adjusted using the Shaffer method, and multiple comparisons were made.

To clarify the differences in health literacy scores, the measurements were compared using the Mann-Whitney U test, dividing the participants into two groups: the low health literacy group, with scores of <33 on the standard index, and the high health literacy group, with scores of 33 or more, which were classified as “insufficient” or “somewhat insufficient” and “sufficient” or “somewhat sufficient,” respectively. The relationship between health literacy and other measurements was examined using Spearman's rank correlation coefficients.

Multiple logistic regression analysis was conducted using binary data for high and low health statuses as dependent variables. The QOL values in this analysis were calculated as a hundredfold value, and the odds ratio was adjusted.

All analyses were performed using the IBM Corp. Released 2016. IBM SPSS Statistics for Windows, Version 25.0. Armonk, NY: IBM Corp. The significance level was set at 5%.

## Results

**Figure 1:**
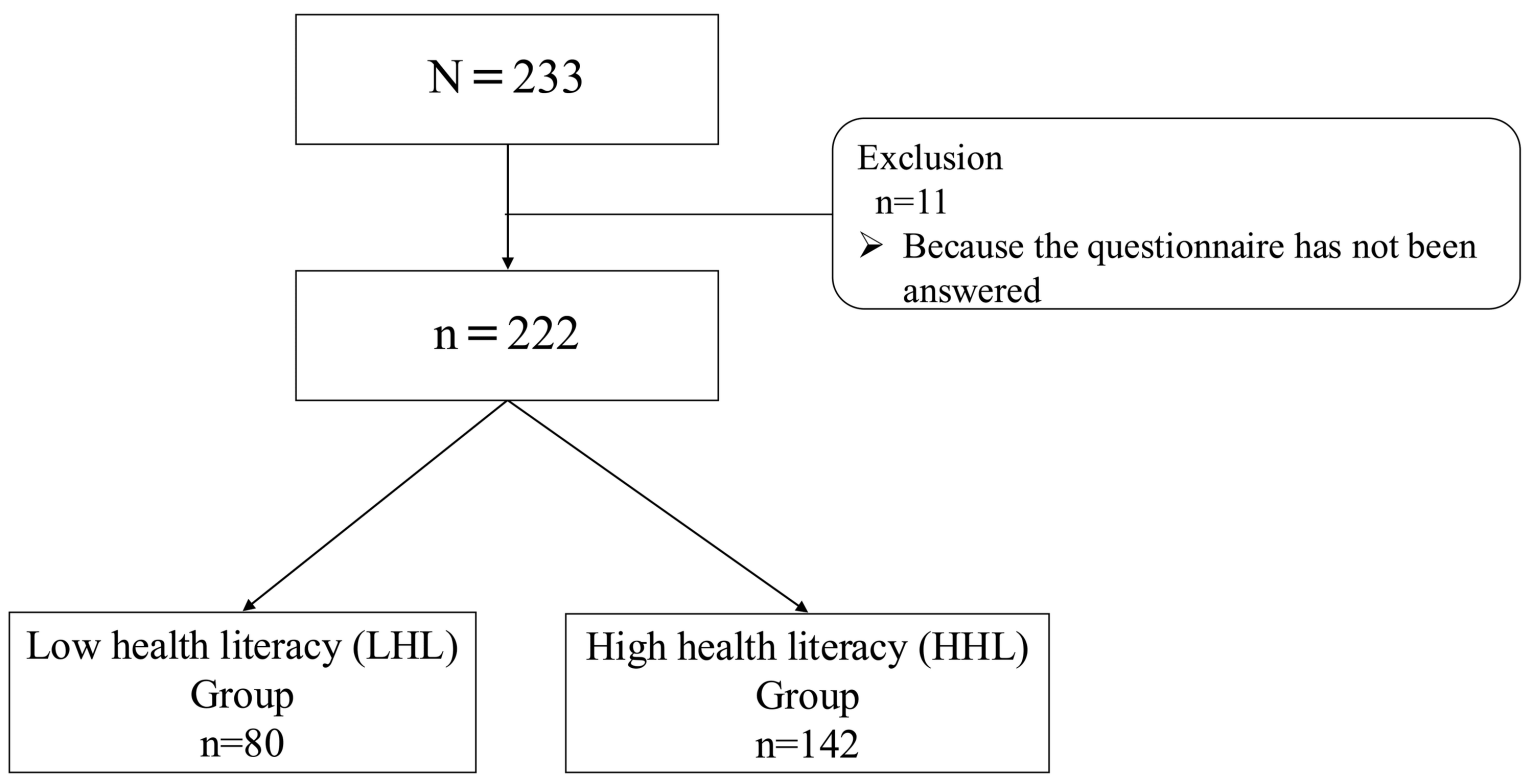
Participants tree

Initially, 233 participants were registered; however, 11 did not complete the questionnaire and were then excluded from the analysis. No subjects met the other exclusion criteria. Consequently, the analysis was conducted on 222 participants (Figure 1). Of these, 142 people with a score of ≥33 on the HLS-EU-Q16 were categorized into the high health literacy group (HHL group), and 80 people with a score of < 33 into the low health literacy group (LHL group).

Table 1 shows the measurement results for all participants and by sex. All results are presented as the median (interquartile range). Women were significantly older than men, had lower body weights and BMIs, and had higher body fat percentages. Health literacy and the B ratio were significantly higher in women than in men. In terms of autonomic nervous activity, women had significantly lower SDNN, RMSSD, LF, and HF values than men.

**Table 1:** The results of the measurement value overall and by gender as median (interquartile range) All data are shown as the median (interquartile range). Using the Mann-Whitney test comparison between men and women: **p < 0.01, *p < 0.05 BMI: body mass index, %BF: percent of body fat, SMI: skeletal muscle mass, HL: health literacy, HHL: high health literacy, LHL: low health literacy, SDNN: standard deviation of the normal and normal interval, RMSSD: square root of the mean of the sum of the square of differences between adjacent NN intervals, TP: total power, LF: low frequency, HL: high frequency

	All (n＝222)		Men (n=59)		Women (n=163)	
age (y)	63.58	[52.75 - 75.00]	57.10	[43.00-71.00]	65.93^**^	[57.00-76.00]
Body weight (kg)	53.10	[47.70 - 62.05]	64.85	[58.75-73.98]	49.80^**^	[46.30-55.10]
BMI (kg/m2)	21.60	[19.85 - 23.70]	22.90	[21.00-24.58]	21.20^**^	[19.40-23.40]
%BF (%)	27.00	[22.60 - 32.85]	22.60	[21.00-24.58]	29.40^**^	[24.70-33.80]
SMI (kg/m2)	6.10	[5.55 - 6.90]	7.60	[7.08-8.10]	5.80^**^	[5.40-6.20]
EQ-5D-5L	0.90	[0.82 - 1.00]	0.867	[0.823-0.921]	0.895	[0.823-1.000]
HL	36.45	[30.21 - 42.71]	32.26	[26.82-37.86]	37.78^**^	[32.29-44.44]
B ratio (b/a)	-0.62	[-0.72 - -0.51]	-0.700	[-0.805--0.580]	-0.610^**^	[-0.690--0.490]
SDNN	32.34	[22.23-51.93]	40.02	[25.13-62.27]	29.70^*^	[21.52-48.14]
RMSSD	26.00	[16.38-42.48]	29.24	[19.88-60.83]	23.90^*^	[14.61-39.17]
LF	43.38	[18.96-157.59]	99.00	[27.73-213.31]	37.34^*^	[16.44-121.51]
HF	93.22	[32.06-235.13]	202.39	[72.90-352.82]	69.41^**^	[28.79-169.02]
LF/HF	0.56	[0.27 - 1.41]	0.497	[0.264-1.441]	0.589	[0.278-1.391]

Table 2 presents the results for the overall and sex-specific health groups. Comparing the overall LHL and HHL groups, the HHL group had significantly higher age and QOL values than the LHL group. There was no difference in the B ratio between the sexes. Among men, the HHL group had significantly higher body fat percentage, RMSSD, and HF than the LHL group. Among women, the HHL group had significantly higher age, weight, BMI, body fat percentage, SMI, and QOL values than the LHL group.

**Table 2:** The results of group comparisons of health literacy overall and by gender as median (interquartile range) All data are shown as the median (interquartile range). Using the Mann-Whitney test, the comparison between the LHL group and the HHL group is **p < 0.01, *p < 0.05. BMI: body mass index, %BF: percent of body fat, SMI: skeletal muscle mass, HL: health literacy, HHL: high health literacy, LHL: low health literacy, SDNN: standard deviation of the normal and normal interval, RMSSD: square root of the mean of the sum of the square of differences between adjacent NN intervals, TP: total power, LF: low frequency, HL: high frequency

	All (n＝222)				Men (n=59)				Women (n=163)			
	LHL group (n=80)		HHL group (n=142)		LHL group (n=33)		HHL group (n=26)		LHL group (n=47)		HHL group (n=116)	
age (y)	58.93	[47.00-73.00]	66.20^**^	[57.00-76.00]	57.88	[43.00-71.50]	56.12	[41.75-70.00]	59.66	[49.00−74.00]	68.47^**^	[64.00-77.00]
Body weight (kg)	52.70	[46.60-64.40]	53.20	[48.30-60.25]	64.60	[59.85-74.05]	65.10	[57.90-73.05]	48.60	[44.78-51.53]	50.40^**^	[47.25-56.30]
BMI (kg/m2)	21.00	[19.50-23.40]	21.85	[20.00-24.00]	22.70	[20.85-24.40]	23.10	[21.40-24.85]	20.50	[19.00-22.13]	21.60^**^	[19.55-23.95]
%BF (%)	23.70	[19.90-29.40]	28.80	[24.25-33.58]	20.00	[18.15−23.75]	24.30^*^	[19.90-27.45]	27.60	[22.38-32.10]	30.20^*^	[25.70-34.40]
SMI (kg/m2)	5.90	[5.50-7.50]	6.10	[5.60-6.80]	7.8	[7.15-8.15]	7.50	[6.95-8.05]	5.60	[5.18-5.90]	5.90^**^	[5.45-6.30]
EQ-5D-5L	0.84	[0.78-0.89]	70.00^**^	[0.83-1.00]	0.867	[0.820-0.895]	0.895	[0.827-1.000]	0.827	[0.718-0.895]	0.895^**^	[0.837-1.000]
HL	27.08	[23.08-30.21]	40.63^**^	[36.46-44.90]	28.13	[23.64-31.25]	38.10^**^	[35.73-41.67]	26.88	[22.86-30.21]	41.67^**^	[36.46-45.83]
B ratio (b/a)	-0.63	[0.75--0.51]	-0.62	[-0.71--0.51]	-0.700	[-0.775--0.580]	-0.700	[-0.830--0.585]	-0.615	[-0.703--0.478]	-0.600	[-0.690--0.500]
SDNN	32.77	[22.09-51.36]	31.63	[22.23-52.56]	36.86	[21.89-61.13]	41.39	[30.13-80.04]	30.89	[19.28-41.06]	29.56	[21.55-49.18]
RMSSD	24.01	[14.98-38.81]	26.64	[16.84-46.47]	26.09	[14.61-51.71]	32.46^*^	[26.52-96.73]	20.79	[13.00-37.72]	24.38	[15.09-40.49]
LF	47.92	[18.27-145.34]	42.95	[19.84-162.17]	64.07	[18.36-208.06]	125.21	[39.02-216.01]	31.06	[16.36-107.05]	38.86	[16.13-136.32]
HF	80.98	[25.92-207.99]	99.49	[37.90-252.14]	104.58	[26.59-337.04]	259.82^**^	[101.50-397.82]	62.55	[24.23-146.64]	70.81	[29.52-169.87]
LF/HF	0.58	[0.28-1.43]	0.54	[0.24-1.40]	0.677	[0.282-1.871]	0.458	[0.197-0.855]	0.571	[0.284-1.036]	0.644	[0.258-1.477]

Table 3 shows the comparative results by age group. There was a tendency for the accelerated pulse wave and autonomic nerve activity to decline with age; in particular, there was a significant decline in older adults aged ≥80 years. There were no significant differences in QOL values according to age group. There was a tendency for health literacy to increase with age, and although a significant difference was found in the Kruskal-Wallis test, no significant difference was found between the groups.

**Table 3:** The results of the measurements by age group as median (interquartile range) All data are shown as the median (interquartile range). Comparison between < 40 years and 40 ages, 50 ages, 60 ages, 70 ages, or ≧ 80 years: **p < 0.01, *p < 0.05; 40 ages and 50 ages, 60 ages, 70 ages, or ≧ 80 years: ##p < 0.01, #p < 0.05; 50 ages and 60 ages, 70 ages, or ≧ 80 years: ++p < 0.01, +p < 0.05; 60 ages and 70 ages, or ≧ 80 years: ♭♭p < 0.01, ♭p < 0.05; 70 ages and ≧ 80 years: §§p < 0.01, §p < 0.05 BMI: body mass index, %BF: percent of body fat, SMI: skeletal muscle mass, HL: health literacy, SDNN: standard deviation of the normal and normal interval, RMSSD: square root of the mean of the sum of the square of differences between adjacent NN intervals, TP: total power, LF: low frequency, HF: high frequency

	＜40 years (n=21)		40 ages(n=23)		50 ages(n=29)		60 ages(n=44)		70 ages(n=81)		≧ 80 years(n=24)		Kruskal Wallis test
age (y)	32.00	[25.00-36.50]	47.00^**^	[43.00-48.25]	54.00^**##^	[51.25-55.00]	66.00^**##++^	[64.00-67.00]	74.00^**##++^^♭♭^	[71.00-76.00]	83.00^**##++^^♭♭§§^	[80.75-86.25]	p < 0.01
Body weight (kg)	55.00	[47.75-65.80]	63.10	[52.38-71.83]	53.80	[44.53-69.33]	55.40	[50.80-60.60]	50.20^#^	[47.15-59.15]	48.10^*##^^♭♭^^§^	[45.03-49.85]	p < 0.01
BMI (kg/m2)	21.80	[19.15-22.20]	22.30	[20.58-23.93]	21.40	[18.40-24.45]	22.30	[20.70-24.10]	21.50	[20.00-23.70]	21.00	[18.90-22.48]	n.s.
%BF (%)	21.80	[17.95-26.70]	24.85	[20.60-28.30]	23.80	[19.63-30.88]	29.70^*^	[23.70-35.80]	29.00^*#^	[25.10-33.45]	28.85	[18.45-32.93]	p < 0.01
SMI (kg/m2)	6.70	[5.65-7.40]	7.20	[5.88-8.10]	5.90	[5.40-7.35]	6.30	[5.80-6.80]	5.90	[5.50-6.55]	5.55	[4.85-6.03]	n.s.
EQ-5D-5L	0.867	[0.845-0.947]	0.895	[0.813-1.000]	0.869	[0.808-1.000]	0.867	[0.823-0.895]	0.895	[0.831-1.000]	0.869	[0.752-1.000]	n.s.
HL	33.33	[28.40-42.71]	30.73	[26.82-40.89]	36.68	[27.34-38.43]	39.58	[30.21-45.83]	35.56	[31.10-42.88]	40.53	[33.33-47.14]	p < 0.05
B ratio (b/a)	-0.840	[-0.920--0.720]	-0.750^*^	[-0.863--0.608]	-0.640^**^	[-0.740--0.505]	-0.620^**^	[-0.700--0.530]	-0.600^**##^	[-0.670--0.495]	-0.540^**##^	[-0.643--0.410]	p < 0.01
SDNN	45.68	[40.60-71.35]	49.83	[35.51-63.76]	42.24	[28.51-54.80]	27.50^**##^	[21.63-32.11]	28.50^**#++^	[20.04-49.01]	21.71^**#^	[15.43-49.48]	p < 0.01
RMSSD	30.83	[21.91-76.66]	37.53	[21.22-46.21]	26.32	[19.53-40.35]	18.54^*##^	[13.85-27.79]	26.55	[15.73-49.73]	24.08	[12.41-46.96]	p < 0.01
LF	109.95	[63.82-196.85]	192.48	[97.51-533.79]	60.34	[37.38-489.41]	25.18^**##++^	[12.10-71.94]	38.09^**##^	[19.41-118.69]	17.48^**##++§§^	[7.60-43.10]	p < 0.01
HF	204.38	[75.84-433.20]	208.51	[99.48-399.41]	125.21	[55.81-501.98]	56.93^**##^	[27.33-111.45]	84.77^**##^	[26.05-192.52]	44.94^**^	[18.54-223.53]	p < 0.01
LF/HF	0.607	[0.311-1.044]	0.610	[0.315-2.014]	0.630	[0.340-1.953]	0.419	[0.273-0.174]	0.657	[0.248-1.719]	0.397	[0.159-0.934]	n.s.

Table 4 shows the correlation coefficients between health literacy and each measurement value. A significantly weak correlation was found between age, BMI, body fat percentage, QOL, and B ratio.

**Table 4:** The results of the correlation coefficient by Spearman's rank correlation coefficient **p < 0.01, *p < 0.05 BMI: body mass index, %BF: percent of body fat, SMI: skeletal muscle mass, SDNN: standard deviation of the normal and normal interval, RMSSD: square root of the mean of the sum of the square of differences between adjacent NN intervals, TP: total power, LF: low frequency, HF: high frequency, HL: health literacy

	HL
age (y)	0.184**
Body weight (kg)	0.002
BMI (kg/m2)	0.140*
%BF (%)	0.271**
SMI (kg/m2)	-0.042
EQ-5D-5L	0.231**
B ratio (b/a)	0.138*
SDNN	0.021
RMSSD	0.075
LF	-0.027
HF	0.089
LF/HF	-0.09

Table 5 shows the results of the multiple logistic regression analysis. The dependent variables in the multiple logistic regression analysis were binary data for the HHL and LHL groups, and the independent variables were age, QOL score, RMSSD, SMI, and B ratio. Variables were entered using the variable increase method based on the likelihood ratio. The correlation between the independent variables was confirmed in advance using Spearman's rank correlation coefficient, and it was confirmed that there was no strong correlation (r=0.8 or more). Age, RMSSD, and QOL were selected as significant independent variables, and high suitability was confirmed with a model chi-square test of p<0.001 and a Hosmer-Lemeshow test of p=0.649.

**Table 5:** Results of the multiple logistic regression analysis RMSSD: square root of the mean of the sum of the square of differences between adjacent NN intervals; QOL: quality of life; SMI: skeletal muscle index

Adopted independent variable	Partial regression coefficient	p-value	Odds ratio	95% CI
Age	0.034	< 0.001	1.034	1.012-1.056
QOL	0.034	< 0.001	1.035	1.015-1.055
RMSSD	0.009	0.048	1.009	1.000-1.018
Model chi-square test: p<0.001, Hosmer-Lemeshow test: p＝0.649				
Dependent variable: High health literacy group = 2, Low health literacy group = 1				
Independent variables not in equation: SMI, B ratio				

## Discussion

Of the participants, 26.6% were men, and 73.4% were women; many of the female participants were older. The median age of the female participants was 13 years higher than that of the male participants, and it is believed that the sex differences in this study were mainly due to age. Moreover, when comparing the ages of the HHL and LHL groups, the HHL group was significantly older, and even among female participants only, the HHL group was significantly older. Multiple logistic regression analysis was performed to identify factors influencing the level of health literacy. Age, RMSSD, and QOL were identified as related factors. The RMSSD reflects the regulation of heart rate by the parasympathetic nervous system [21]. Our results showed that the odds of being classified as having HHL increased with age and higher RMSSD and QOL. Older people have low levels of health literacy, which decline with age [22-24], and there are reports that cognitive function is mainly related to the decline in health literacy with age [23,25]. Furthermore, there are reports that people with high literacy are more likely to be older, have a higher level of education, rate their health as good, have specific chronic diseases [26], actively engage with medical professionals, and have a high ability to understand medical information [27]. Sex is also related to a decline in health literacy among the elderly, and there are reports that women generally have higher health literacy than men [28,29]. The older participants in this study were recruited from among those who attended a local medical fitness center; therefore, we might probably gather a large number of older women with a high level of health literacy. Therefore, this group might have a higher HL level of health literacy than the general community population. However, a new finding of this study is that RMSSD, which is one of the indicators of autonomic nervous activity, is related to health literacy. In this preliminary cross-sectional study, we confirmed the relationship between these factors. However, further longitudinal and intervention studies are needed to clarify whether parasympathetic nervous system activity affects health literacy. Because age affects health literacy, it is important to study how health literacy impacts the parasympathetic nervous system in different age groups over time. Furthermore, this study found that health literacy was high in the older age group and low in the younger age group. To prevent lifestyle-related diseases, which often occur in middle-aged people before old age, there is a need for health literacy interventions at a younger age.

The ratio of b-wave height to a-wave height, as measured using acceleration plethysmography (B ratio, b/a), reflects the progression of arteriosclerosis and increases with age [30]. In the present study, the B ratio increased significantly with age. When comparing men and women, the B ratio was higher in women than in men. This may be because there were more older women than men in this study. Regarding the relationship with health literacy, there was no significant difference between HHL and LHL, and a weak but significant correlation was found between health literacy and the B ratio. Considering the possibility of an alpha error owing to the large sample size, it is believed that there is no relationship between health literacy and acceleration pulse waves in the results of this study.

This study had several limitations. First, this was a cross-sectional study, and the causal relationship between health literacy-related factors remains unclear. Future longitudinal investigations of the health-related factors found in this study are warranted. Second, because age and sex were considered confounding factors related to health literacy, we compared and tested them separately for men and women. The age range covered a wide range, from the 20s to the 90s. Therefore, we conducted separate analyses for men and women and comparisons by age group. However, when the data were analyzed by age and sex, the number of people in each group decreased. Therefore, comparisons by age group were limited to mixed-sex comparisons. In this comparison, there were more women, particularly in older age groups, which may have affected the results. Thus, it is necessary to increase the number of participants in the future to conduct further verification. Third, participants in this study were recruited from among medical university staff, attendees at local health awareness events, and users of commercial gymnasiums; therefore, it is possible that many of them had higher levels of health literacy and were more health-conscious than the average adult living in the area, which may have affected our results in terms of generalizability, and further verification with different subjects is required. Another limitation is that it does not take into account personal factors such as education and employment history, which are thought to influence health literacy. Since education in Japan is compulsory up to junior high school, the effect of educational status is thought to be small, but it will be necessary to take these individual factors into account in the future. However, despite its various limitations, this study is useful because it identified factors related to health literacy among Japanese adults of a wide range of ages. Therefore, it is necessary to focus on health literacy from a younger age and intervene. Moreover, we will focus on autonomic nervous system activity from an early stage, particularly before the onset of fatal diseases, and verify this longitudinally in the future.

## Conclusions

This study investigated whether acceleration plethysmography and autonomic nervous system activity are associated with health literacy in Japanese adults. These results suggest that parasympathetic nervous system activity, age, and quality of life may be associated with health literacy. In the future, these findings suggest the need to investigate the relationship between health literacy and autonomic nervous system activity in the general adult population in Japan and to clarify the causal relationship between these factors and health maintenance. Furthermore, there is a tendency for health literacy to decline among younger people; therefore, there is a need for health education to improve health literacy in younger people. To further verify the findings of this study, it is important to conduct longitudinal observational and intervention studies.
